# Subgroup Analyses of Girl2Girl, a Text Messaging-Based Teen Pregnancy Prevention Program for Sexual Minority Girls: Results from a National RCT

**DOI:** 10.1007/s11121-023-01493-6

**Published:** 2023-02-08

**Authors:** Michele L. Ybarra, Elizabeth Saewyc, Margaret Rosario, Shira Dunsiger

**Affiliations:** 1 Center for Innovative Public Health Research, 555 El Camino Real #A347, CA San Clemente, 92672 USA; 2 University of British Columbia School of Nursing, Vancouver, Canada; 3 The City University of New York – City College and Graduate Center, New York, NY USA; 4 Brown University, Providence, RI USA

**Keywords:** LGB+ pregnancy prevention, Text messaging, RCT, Sub-group analyses

## Abstract

This study aims to investigate whether Girl2Girl, a text messaging-based pregnancy prevention program for cisgender LGB+ girls, had different effects on subgroups based on age, sexual identity, and experience with penile-vaginal sex. A total of 948 girls, 14–18 years old, were recruited nationally via social media and enrolled over the telephone. Once they completed the baseline, they were randomized to either Girl2Girl or an attention-matched control program that discussed “healthy lifestyle” topics (e.g., self-esteem). Both programs were 5 months long: Girls received daily messages for 8 weeks, and then went through a “latent” period of 3 months, and finished with a 1-week review. Outcome measures included condom-protected sex, uptake of other types of birth control, abstinence, and pregnancy. Measures were collected at baseline; 3-month, 6-month, 9-month, and 12-month post-intervention end, which was 17 months after enrollment. Effect modification was examined using longitudinal mixed effects models. Overall, results suggested significant moderating effects of age, *(f*^2^ = .12), sexual identity (*f*^2^ < .14), and sexual experience (*f*^2^ = .11) on rates of condom use and use of other contraception. Although there were no significant moderating effects on pregnancy, abstinence, or intentions to use condoms, use birth control, or be abstinent, (*p*’s > .16), patterns of effects were in the same direction as for significant findings. For example, at 9-month post-intervention, among those who identified as bisexual, the incidence rate of protected sex events was 39% higher for intervention vs. control (IRR = 1.39, 95% CI: 1.06–2.70), adjusting for baseline rate of condom use and sexual experience. Similarly, at 12 months, among bisexual participants, intervention participants had a significantly higher IRR of condom-protected sexual events (IRR = 2.65, 95% CI: 1.31–5.34). There were also higher odds of uptake of birth control use other than condoms for intervention vs. control at 6- (OR = 1.10, 95% CI: 1.01–1.77), 9 m (OR = 1.11, 95% CI: 1.07–1.89), and 12-month (OR = 1.13, 95% CI: 1.07–1.78) follow-up. Girl2Girl appears to be particularly effective for older adolescents, bisexual girls, and those who have already had penile-vaginal sex. No one single approach is going to affect teen pregnancy. Instead, it is more likely that different intervention content and delivery methods will be more accessible and salient to some but not other youth. Understanding for whom the intervention works is just as important as understanding for whom the intervention does not, as this can inform opportunities for future intervention development.

Clinical Trial Registration: ClinicalTrials.gov ID# NCT03029962.

The teenage birth rate has been steadily falling for the past three decades (Martin et al., 2021). In 2019, 16.7 births per 1000 adolescents assigned female at birth were recorded (CDC, 2021). Nonetheless, birth rates are the highest in the USA compared to other similarly resourced countries (Sedgh et al., 2015), and disparities in birth rates persist, including by race, ethnicity, and household income (Romero et al., 2016). Although not often recognized, disparate rates by sexual identity also are noted. Studies consistently find that sexual minority cisgender girls, those who are lesbian, gay, bisexual, or other sexual minority identities, collectively referred to as LGB+ herein, are significantly more likely to be pregnant in adolescence than heterosexual girls (Goodenow et al., 2008; Saewyc et al., 1999; Tornello et al., 2014). At the same time, youth who are sexual minority lack relevant and inclusive sex education (Rabbitte, 2020). Efforts to improve access to evidence-based pregnancy prevention for LGB+ girls could have public health significance, particularly as the number of young people who identify as sexual minority appears to be increasing (Phillips et al., 2019).

Universal interventions endeavor to affect the behavior of all youth. It is important to examine the impact that a program may have on important subgroups of youth as this has implications for future adaptations and intervention development. Indeed, previous work has noted differences by gender and ethnicity: Kirby et al. (2004) found differences in the effects of a sexual risk reduction program for 9th graders by gender, ethnicity, and sexual activity at baseline. Boys had higher rates of condom use than girls. Hispanic youth were more likely to use a condom than non-Hispanic youth. Youth who were sexually active at baseline were more likely to use condoms than youth who started having sex during the study. A pregnancy prevention program delivered in Boys and Girls Clubs was tested for cost effectiveness as well as the additional effect of a text messaging enhancement (Bull et al., 2016). Although differences in behavioral outcomes were not detected among all youth, the intervention appeared to have an effect on pregnancy among Hispanic youth. Together, these studies suggest that subgroup analyses can reveal for whom the intervention may work better.

Teen pregnancy prevention programs targeted to LGB+ girls need to take into account and affect the behavior of important subgroups: Research consistently shows that youth who identify as bisexual, as compared to lesbian or gay, have higher rates of sexual behaviors that increase their risk for sexually transmitted infections and other negative sexual health outcomes (Rosario et al., 2020; White Hughto et al., 2016). To affect disparate pregnancy rates then, an LGB+ teen pregnancy prevention program needs to ensure that bisexual girls’ behavior is being affected at least as much as that of lesbian girls, if not more. Similarly, those who are having sex are at more immediate risk for pregnancy and other outcomes targeted by a sexual health program compared to those who have not started having sex. While it is important to include sexually inexperienced youth in universal programs to arm them with the skills they will need to make healthy choices the first time, and every time, they have sex; it is equally important to be sure that the program is sufficiently and specifically affecting the behavior of sexually experienced youth. Finally, understanding how age is related to intervention impact could inform future adaptations. Perhaps the content is written too “young,” such that older youth do not resonate with the program messages. On the other hand, perhaps the delivery modality, which is text messaging in the current investigation, requires a certain level of reading comprehension to intuit how to apply written words to one’s real life.

To address the lack of sexual minority-inclusive teen pregnancy prevention programming, we developed and tested Girl2Girl, an mHealth pregnancy prevention program tailored to the unique needs of sexually experienced and inexperienced LGB+ adolescent girls. Participants received between 4 and 12 messages per day for 7 weeks. Girls then experienced a “latency” period of 12 weeks when they received 1–2 messages per week but were otherwise not contacted. A 1-week “booster” that reinforced the main program messages was delivered at the end of the latency period. The total intervention period was 5 months.

Messages were written to address the components of the Information-Motivation-Behavioral Skills (IMB) model (Fisher & Fisher, 1992, 2000), including socio-cultural factors, such as aspects of healthy relationships. Girls were assigned to one of four “paths” of messages based upon their sexual experience (ever versus never having had sex) and sexual identity (i.e., exclusively lesbian/gay versus all others). Of exception were girls who identified as queer because some girls use it in a way that aligns with a lesbian identity, and others, with a bisexual identity (Ybarra et al., 2019). In this case, their sexual attractions determined which content they received.

Each Girl2Girl intervention participant also was paired with another participant, her “Text Buddy.” Buddies were encouraged to text with each other about what they were learning in the program. Messages were routed through the study server so that messages could be monitored and individuals’ phone numbers could be kept private.

The control arm received a similar number of messages for the same number of days as the intervention group. Content addressed topics relevant to adolescents, such as self-esteem and how to deal with bullying. To help blind this arm, 2 days of pregnancy prevention content readily available online was included. Interactive components, including Text Buddy, Badges (i.e., girls earned a “badge” for attaining pre-set goals, such as getting tested for HIV, getting condoms), and other two-way messages, were not available to the control group.

At intervention end, 5 months after girls were enrolled, youth randomly assigned to the intervention arm reported higher rates of condom use and use of other types of birth control (Ybarra et al., 2021). Behavior change persisted through 12-month follow-up (Ybarra et al., 2023). Here, we aim to understand for whom Girl2Girl might have a stronger impact. Analyses focus on subgroups defined by baseline age, sexual orientation, and penile-vaginal sex experience. Implications for future technology-based teen pregnancy prevention programs are explored and suggestions for intervention design that may promote greater generalizability of impact across cisgender LGB+ adolescents are provided.

## Methods

The study is an individual-level randomized controlled trial (RCT). Cisgender girls were assigned either to Girl2Girl, a text messaging-based teen pregnancy prevention program, or a “healthy lifestyle” control program. Greater detail about the intervention can be found elsewhere (Ybarra et al., 2021). A total of 948 girls participated in the RCT.

Advarra Institutional Review Board, an Office of Human Research Protections (OHRP)-approved IRB, reviewed and approved the research protocol. We were granted a waiver of parental permission so that girls who wished to participate would not have to put themselves in a potentially unsafe situation by disclosing their sexual identity to their parents (Ybarra et al., 2016a, b).

### Participants

Girls were recruited across the USA. Eligible participants were (1) aged 14 to 18 years, (2) cisgender (i.e., assigned female sex at birth and endorsed a female gender identity), (3) sexual minority (i.e., had a sexual identity other than heterosexual), (4) had not yet graduated high school, (5) English speaking, (6) owned their own cell phone and had an unlimited text messaging plan, (7) intended to have the same cell phone number for 12 months, (8) had used text messaging for at least 6 months, and (9) were able to provide informed assent for those under 18 years of age and informed consent for those 18 years of age, including the capacity to assent/consent and to pass a self-safety assessment. Those who knew another girl in the RCT or had participated in an intervention development activity (e.g., focus groups) were excluded.

### Recruitment and Retention Procedures

Girls were recruited and enrolled between January 23, 2017, and January 12, 2018. Participants were recruited primarily through Facebook and Instagram. Advertisements targeted users with profiles who indicated they were female, between 14 and 18 years of age, and “interested in females” or “interested in males and females.”

Interested youth clicked on the advertisement, which linked them to the online eligibility screening form. Girls who appeared to be eligible were contacted sequentially via the telephone to confirm eligibility and fully discuss study risks, given the waiver of parental permission. Youth living in rural settings or identifying as racial/ethnic minority were given preference to ensure a demographically diverse sample.

Randomization. After completing the baseline survey, girls were randomized to treatment (*n* = 473) or control groups (*n* = 475) at a one-to-one randomization allocation ratio (Ybarra et al., 2021). Participants were stratified by sexual experience and sexual identity. Participants, but not researchers, were blind to arm allocation.

Data Collection. Surveys were collected online and via text messaging. Girls completed assessments at baseline; 7 weeks later; intervention end, 5 months later; and 3-, 6-, 9-, and 12-month post-intervention end. Data at 6-, 9-, and 12-month post-intervention end are analyzed in the current investigation.

Incentive. Youth were not incentivized to complete the baseline survey, but were incentivized to complete follow-up surveys. Youth were asked to complete both online (longer) and text messaging (shorter) surveys. The former were incentivized at a higher rate to reflect the greater burden. Incentives also generally increased over time to attend to potential waning of interest in continued participation. Due to funding uncertainties mid-way through the RCT (Kay, 2017; Uzzell & Troiano, 2019), the survey mode and incentive structure changed. As such, participants received between $5 and 25 for completing the survey at intervention end based upon whether they completed the survey via text or online. Incentives for 6-month post-intervention end survey were $30–35; 9-month post-intervention end, $10–40; and 12-month post-intervention end, $10–45.

### Measures

Main outcomes included (1) condom-protected penile-vaginal sex in the past 3 months (operationalized as a count of number of protected sex events), (2) current use of contraception other than condoms (operationalized as a binary indicator of current use), (3) abstinence from penile-vaginal sex in the past 3 months (operationalized as a binary indicator of abstinence), and (4) pregnancy (operationalized as a binary indicator of pregnancy since last contact). Measures were collected at baseline, 3-month, 6-month, 9-month, and 12-month post-intervention end. Data from 6-, 9-, and 12-month follow-ups were analyzed in the current investigation. Outcome measures were adapted from those recommended by the Office of Adolescent Health (Office of Adolescent Health, 2014).

### Statistical Analyses

Associations between intervention condition and primary outcomes were estimated; namely, number of protected sex events, birth control use other than condoms, abstinence, and pregnancy rates. *T*-tests (for continuous variables), chi-squared tests (for categorical variables), and non-parametrics, as appropriate, were used to identify statistical confounders of the intervention effect, as defined by variables that were statistically associated with both the arm assignment and outcome at a *p* < 0.10 level. Next, using a series of longitudinal mixed effects models, outcomes were regressed on group (i.e., intervention vs. control), time, group × time, as well as potential confounders. Time was operationalized by assessment number (e.g., 6 month). Models also included the main effects of the potential moderator: age (younger vs. older), sexual identity (lesbian vs. bisexual), and sexual experience (had penile-vaginal sex vs. did not have such experience). They also included the interaction between the moderator × time for each pregnancy prevention outcome, moderator × group, and moderator × group × time. Models included a subject-specific intercept to account for repeated measurements within participant over time. As sexual experience may vary over time, it was included as a time-varying covariate in the model.

One advantage of mixed effects models is their flexibility with regard to the outcome distribution, meaning they can be used for outcomes that follow normal, negative binomial, and Bernoulli distributions (Zhang & Davidian, 2001). A variable was considered a moderator if the effect of moderator × group was statistically different from 0. Effect size estimates (*f*^2^) were calculated to help both in articulating the magnitude of the effect but also in comparing effects across models. Medium effect size would correspond to *f*^2^ = 0.15 (Cohen, 2013). Odds ratios/relative risks, adjusted for baseline levels of the outcome of interest, and sexual experience where applicable were then estimated (along with 95% confidence intervals) to show the magnitude and direction of the effect.

**Fig. 1:**
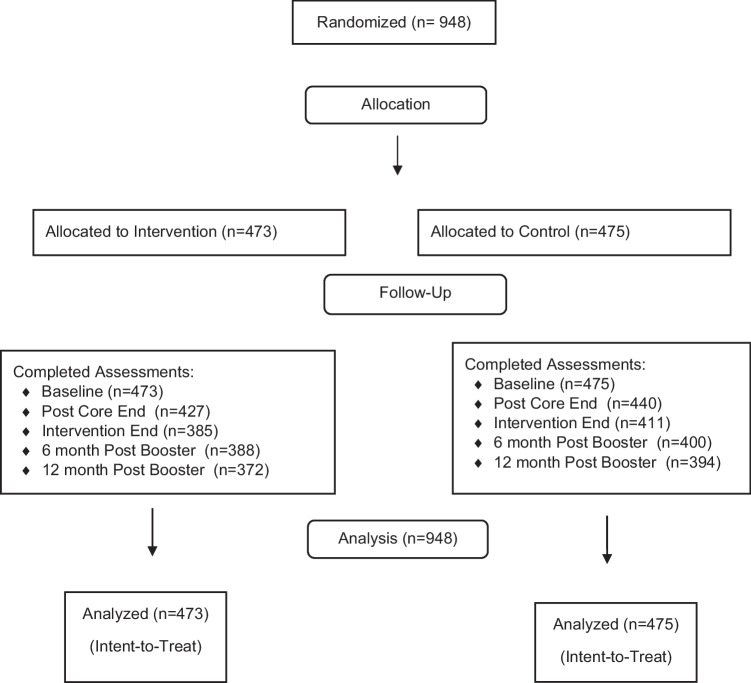
CONSORT Table

All analyses were run on the intent to treat sample (Fig. 1). Models use a likelihood-based approach to estimation, making use of all available data without directly imputing missing data. This allows for regression estimates that are consistent, even with missing data (Kiefer & Wolfowitz, 1956). All analyses were run in SAS 9.3, and significance level was set at 0.05 a priori.

## Results

By study design, almost all participants (*n* = 937, 99%) identified as cisgender female. Participants ranged in age from 14 to 18 years, with a mean of 16.1 years at study initiation (SD = 1.2). Almost one in two (45%) participants identified as lesbian. Nearly a quarter (24%) identified as Hispanic. About half (55%) identified as White race, 14% as Black/African American, and 15% as mixed race. One in four (27%) appraised their household income to be lower than the average household. Two in three (67%) had ever had penile-vaginal sex, and one in three (24%) were currently on birth control. Four percent had ever been pregnant.

At 12-month follow-up, 79% of lesbian and 83% of bisexual girls (*p* > 0.05), and 92% of younger and 90% of older girls completed the survey. Far fewer lesbian (28%) than bisexual (58%) girls reported penile-vaginal sex (*p* < 0.01); rates were similar for younger (30%) and older (35%, *p* > 0.05) girls.

Overall, results suggested significant moderating effects of age, sexual identity, and sexual experience on rates of condom use and use of other contraception. Although there were no significant moderating effects on abstinence, pregnancy, or intentions (*p* > 0.16), patterns of effects were in the same direction as for significant findings.

A summary table of subgroup effect sizes is provided in Table 1. Specifically, models indicated that age was a significant moderator of the intervention effect on rates of condom-protected sexual act (*p* = 0.001). At 9-month post-intervention end, 14 months after youth enrolled in the program; among those 16–18 years of age, the incidence rate of protected sex events was 111% higher for intervention vs. control (IRR = 2.11, 95% CI: 1.18–4.88), adjusting for baseline rate of condom use and sexual experience. In contrast, intervention effects were not statistically significant among younger participants, aged 14–15 years, *p* = 0.16. A similar pattern was seen at 12 months, with IRR of 1.69 among older participants (95% CI: 1.01–2.38) and no intervention effects among younger participants (*p* = 0.98). Effect sizes were in the small-medium range (*f*^2^ = 0.12).

**Table 1:** Subgroup analyses of treatment effects on pregnancy preventive outcomes

	Rate of condom-protected sexual acts (IRR)	Relative odds of contraceptive use other than condoms (OR)	Relative odds of abstinence (OR)	Relative odds of pregnancy (OR)
Age				
14–15 yrs, *n* = 322	6 m: 1.46 (.60–3.59) 9 m: .47 (.21–1.09) 12 m: 1.01 (.49–2.08)	6 m: .59 (.18–1.90) 9 m: .32 (.13–.76) 12 m: .62 (.31–1.23)	6 m: 9.26 (0–13.92) 9 m: .88 (.53–1.47) 12 m: .65 (.39–1.07)	6 m: 2.68 (.24–29.90) 9 m: 1.33 (.08–21.45) 12 m: 2.01 (.33–12.25)
16–18 yrs, *n* = 626	6 m: 1.39 (.86–2.23) 9 m: 2.11 (1.18–4.88) 12 m: 1.69 (1.01–2.38)	6 m: .67 (.31–1.44) 9 m: 1.01 (.63–1.65) 12 m: .90 (.56–1.46)	6 m: 1.88 (.17–13.92) 9 m: .90 (.64–1.27) 12 m: .88 (.62–1.25)	6 m: 1.52 (.49–4.69) 9 m: .40 (.10–1.55) 12 m: .56 (.13–2.36)
Sexual identity				
Bisexual youth, *n* = 397	6 m: 1.37 (.51–3.71) 9 m: 1.39 (1.06–2.70) 12 m: 2.65 (1.31–5.34)	6 m: 1.10 (1.01–1.77) 9 m: 1.11 (1.07–1.89) 12 m: 1.13 (1.07–1.78)	6 m: 9.02 (0–33.19) 9 m: 1.05 (.68–1.62) 12 m: .96 (.62–1.48)	6 m: 1.40 (.23–8.50) 9 m: .15 (.02–1.26) 12 m: .36 (.07–1.91)
Lesbian youth, *n* = 430	6 m: 1.61 (1.11–2.33) 9 m: .84 (.42–1.67) 12 m: 1.05 (.62–1.80)	6 m: .69 (.26–1.35) 9 m: .91 (.57–1.47) 12 m: .89 (.56–1.40)	6 m: 2.31 (.21–25.70) 9 m: .80 (.54–1.18) 12 m: .71 (.48–1.05)	6 m: 2.05 (.59–7.09) 9 m: 1.74 (.28–10.49) 12 m: 2.33 (.42–12.83)
Sexual experience				
Ever had penile-vaginal sex, *n* = 309	6 m: 1.44 (.65–3.22) 9 m: 1.47 (1.07–2.89) 12 m: 2.09 (1.12–3.89)	6 m: .40 (.17–1.93) 9 m: 1.05 (.47–2.29) 12 m: .98 (.48–2.00)	6 m: 13.02 (0–28.02) 9 m: .80 (.47–1.35) 12 m: .84 (.50–1.40)	6 m: 1.71 (.54–5.37) 9 m: .58 (.17–2.04) 12 m: 1.04 (.26–4.26)
Never had penile-vaginal sex, *n* = 636	6 m: 2.89 (.42–5.89) 9 m: .69 (.36–1.31) 12 m: 1.15 (.63–2.07)	6 m: 1.33 (.47–3.82) 9 m: .67 (.42–1.09) 12 m: .72 (.45–1.15)	6 m: 2.113 (.19–23.63) 9 m: .89 (.61–1.30) 12 m: .73 (.50–1.40)	6 m: 2.13 (.19–23.64) 9 m: .99 (0.00–31.59) 12 m: .71 (.12–4.25)

Statistical significance would be indicated by a confidence interval that does not include 1.00

OR/IRR (95% CI) reported for each model. Models are adjusted for baseline levels of the outcome of interest, and sexual experience where applicable

Similarly, sexual experience, which was a binary indicator of ever having penile-vaginal sex, was a moderator of the intervention effects on rates of condom-protected sexual acts (*p* = 0.02). Specifically, at 9-month post-intervention, among those with sexual experience, the incidence rate of protected sex events was 47% higher for intervention vs. control group participants (IRR = 1.47, 95% CI: 1.07–2.89), adjusting for baseline rate of condom use. There were no differences among those with no sexual experience at baseline (*p* = 0.25). Similarly, at 12 months, there were significant differences between intervention and control girls among those who entered the RCT with sexual experience (IRR = 2.09, 95% CI: 1.12–3.89). No effect was detected among those without sexual experience at baseline (*p* = 0.65). Effect sizes were in the small-medium range, *f*^2^ = 0.11.

Finally, sexual identity was a significant moderator of intervention effects in both condom use and contraception use over time, with effects sizes in the small to medium size (*f*^2^ < 0.14). At 9-month post-intervention, among those who identified as bisexual, the incidence rate of protected sex events was 39% higher for intervention vs. control (IRR = 1.39, 95% CI: 1.06–2.70), adjusting for baseline rate of condom use and sexual experience. Similarly, at 12 months, among bisexual participants, intervention participants had a significantly higher relative rate of condom-protected sexual events (IRR = 2.65, 95% CI: 1.31–5.34). There was a similar pattern for bisexual participants at 6- and 12-month follow-up with respect to intervention effects on contraception use over time. Among bisexual participants, there also were higher odds of uptake of birth control use other than condoms for intervention vs. control at 6-month (OR = 1.10, 95% CI: 1.01–1.77), 9-month (OR = 1.11, 95% CI: 1.07–1.89), and 12-month follow-up (OR = 1.13, 95% CI: 1.07–1.78).

No significant effects of the intervention on abstinence or pregnancy were detected (*f*^2^ < 0.08 and *f*^2^ < 0.07 respectively).

## Discussion

Girl2Girl is the first comprehensive mHealth teenage pregnancy prevention intervention developed and tested nationally, as well as one of the first pregnancy prevention programs developed for cisgender sexual minority girls of which we are aware. As previously reported (Ybarra et al., 2021; Ybarra et al., 2023), both short-term and longer-term effects offer compelling evidence that the intervention is associated with increases in condom use and contraception uptake that persist up to 12 months after the intervention ends. The current findings suggest that Girl2Girl is associated with greater behavior change for older girls, girls who have had penile-vaginal sex, and for bisexual girls.

The intervention development was guided by the Information-Motivation-Behavioral Skills model of (HIV) prevention (Fisher & Fisher, 1992, 2005), adapted for pregnancy prevention. It posits that preventive behavior is the result of one’s exposure to information about how to prevent the outcome (i.e., pregnancy, in the current case), motivation to engage in preventive behaviors, and behavioral skills and abilities to enact preventive behavior. Information and motivation are putative causal predictors of the onset and sustainability of one’s preventive behaviors, which are mediated by behavioral skills. Key components of motivation are attitudes towards the target behaviors, as well as subjective norms and behavioral intentions to engage in the target behaviors. Central aspects of behavioral skills include both the ability to perform the behavior (e.g., put on a condom correctly) and one’s self-efficacy to engage in the behavior (Fisher et al., 2006). Social learning theory similarly posits that motivation and motor reproduction (i.e., being able to perform the behavior) are critical to learning a new behavior (Bandura, 1977, 2003; Bandura & Walters, 1963). It further proposes that retention, one’s ability to store in long-term memory how to enact the task; and attention, including the salience of the person modeling the behavior, also contribute to learned behaviors. Together, these mechanisms may help explain why the intervention had a stronger impact for girls who had had sex before they entered the study. Indeed, sexually experienced young women may have had greater self-efficacy in their ability to use condoms and initiate birth control because the intervention messaging builds on their previous sexual experience and reinforces their positive expectations for being able to navigate contraception and barriers. If they can more easily visualize when and how to take protective action than girls who have not yet had sex, they may find it easier to act and to sustain that action. On the other hand, the intervention content might be less compelling and actionable for girls who are tasked with understanding the information in a theoretical form. The hypothetical situation may be harder for sexually inexperienced girls to absorb, retain, and effectively apply to future sexual circumstances that might involve pregnancy risk. These girls may remember the information, but may not be ready for unexpected variations in the “script,” or be sufficiently confident in their ability to communicate their choices to their partners. Future research might explore additional booster sessions for sexually inexperienced girls as they navigate their way through their first sexual experience.

The lack of intervention impact for younger girls could be due to neurocognitive development. Self-control in adolescence is diminished because subcortical limbic systems have greater influence than cortical systems during this time relative to adulthood (Casey, 2015). In addition, among those aged 14–18 years, self-control decreases with youthfulness (Cohen et al., 2016). It may be that planning, particularly around obtaining condoms before the encounter, and exerting self-control, particularly during the sexual encounter to use the condom, is developmentally more achievable for older adolescents.

Differential power in the sexual relationship may be another reason why we see different intervention impacts by age. Miller and colleagues found that younger girls were more likely than older girls to have a larger gap between their age and that of their sexual partner, and they were more likely to report having been coerced or forced to have sex (Miller et al., 2010). To the extent that younger adolescents may have less power in relationships to negotiate sexual decisions such as condom use, or are more vulnerable to force or sexual coercion, this may help explain greater incidence of protected sex and contraceptive use among older girls.

The effect that Girl2Girl had on pregnancy preventive behaviors for lesbian girls is less clear than its effect for bisexual girls. One in four lesbian girls had penile-vaginal sex during the observation period compared to one in two bisexual girls. As such, statistical power may be an issue. Perhaps with a larger sample, significant intervention effects might have been detected for lesbian girls. Importantly, retention rates were similar for lesbian and bisexual girls This suggests that there was not differential drop out because lesbian girls found the content to be less relevant than bisexual girls did, although it is possible that lesbian girls paid less attention to the content over time. Focus groups conducted during the formative phase suggested that some lesbian girls did not anticipate the possibility of, nor desire, penile-vaginal sex (Ybarra et al., 2020), even though some lesbian girls engage in penile-vaginal sex (Rosario et al., 2020; Ybarra et al., 2020). For example, some girls have penile-vaginal sex as a way to explore and confirm their sexual orientation, or as a response to self-imposed or external pressure from others (Rosario et al., 2020). Influences on sexual decision making, as well as the notion that lesbian girls do not typically see themselves at risk for pregnancy, were emphasized in the Girl2Girl content. Future research could test various methods of messaging to identify additionally effective ways of delivering pregnancy prevention information to lesbian girls.

## Limitations

Findings should be interpreted within the study limitations. First, recruitment targeted girls who have their own cell phone and have unlimited text messaging. While this represents 95% of youth (Common Sense Media, 2018), there is an important minority of youth who does not own a phone. Second, girls were recruited through social media. Again, while most young people were on Facebook and Instagram at the time of recruitment, not all youth were; and among those on social media, those who clicked on a social media advertisement were likely different from those who did not. This may nonetheless be reflective of youth who would be more likely to utilize this type of intervention, if it were made publicly available and advertised through social media channels. Finally, gender minority youth were excluded because messages were written from a cisgender assumptive perspective. Future interventions should endeavor to be gender inclusive.

## Conclusion

Girl2Girl appears to be particularly effective for older adolescents, bisexual girls, and those who have already had penile-vaginal sex. No one single approach is going to affect teen pregnancy. Instead, it is more likely that different intervention content and delivery methods will be more accessible and effective for some but not other youth. Understanding for whom the intervention works is just as important as understanding for whom the intervention does not, as this can shape future intervention development to address the target population in a different and more effective manner.

## Data Availability

Data are not publicly available. They may be available upon request.
